# Discovery of a Novel Compound Enhancing SVZ Neurogenic Effects via Human Neural Stem Cell-Based Phenotypic Screening

**DOI:** 10.3390/ph19040536

**Published:** 2026-03-26

**Authors:** Atsushi Nakane, Katsushi Kitahara, Riku Fukushima, Tetsuro Nariai, Kazuto Yamazaki, Hidetaka Nagata

**Affiliations:** Sumitomo Pharma Co., Ltd. 1–98, Kasugade-naka 3-chome, Konohana-ku, Osaka 554-0022, Japan; atsushi.nakane@racthera.co.jp (A.N.); katsushi.kitahara@sumitomo-pharma.co.jp (K.K.); riku.fukushima@sumitomo-pharma.co.jp (R.F.);

**Keywords:** neural stem cells, subventricular zone, neurogenesis, phenotypic screening, adult neurogenesis

## Abstract

**Background/Objectives:** Adult neural stem cells retain the capacity to generate immature neuronal lineages; however, pharmacological approaches that robustly enhance neurogenic activity remain limited. To identify compounds with intrinsic activity under physiologically restrictive conditions, we aimed to screen for small molecules that promote neural stem cell proliferation in the absence of exogenous growth factors and are compatible with central nervous system drug discovery. **Methods**: We developed a human neural stem cell–based phenotypic screening cascade performed under growth factor–free conditions. Compound activity was evaluated in vitro by ATP-based proliferation assays, BrdU incorporation, and assessment of neurogenic marker analysis. In vivo neurogenic effects were assessed in adult rats by BrdU labeling and immunohistochemical analysis of BrdU/Nestin- and BrdU/DCX-positive cells in the subventricular zone and hippocampal subgranular zone, together with pharmacokinetic analysis to assess brain exposure. **Results**: Using this platform, we identified Lead-238 as a small-molecule that enhanced neural stem cell proliferation and neurogenic output in vitro. In vivo, Lead-238 increased neurogenic activity in the subventricular zone, as evidenced by elevated numbers of BrdU-positive, BrdU/DCX-positive, and BrdU/Nestin-positive cells, whereas no detectable effects were observed in the hippocampal subgranular zone. Lead-238 achieved sufficient brain exposure, and its neurogenic effects were not readily explained by established neurogenic pathways. **Conclusions**: These findings demonstrate that growth factor–free phenotypic screening using human neural stem cells is an effective approach for identifying compounds that enhance adult neurogenic activity and identify Lead-238 as a small molecule that increases neurogenic activity in the subventricular zone without detectable effects in the hippocampal subgranular zone.

## 1. Introduction

Neurogenesis is a developmental process defined as the generation and maturation of new functional neurons in the adult central nervous system (CNS). It is well established that the adult mammalian brain harbors neural stem cells (NSCs) in the subventricular zone (SVZ) lining the walls of the lateral ventricles and in the subgranular zone (SGZ) of the hippocampus, where neurogenesis persists throughout life [1,2,3,4]. Within these niches, NSCs are tightly regulated between quiescent and proliferative states [5,6,7,8]. Experimental studies have reported that neurogenesis is influenced not only by physiological factors such as aging, stress, and physical exercise, but also by brain injuries, including seizures, stroke, and traumatic brain injury [9].

Among the factors regulating neural stem cell behavior in the intact adult brain, multiple growth factors, including epidermal growth factor (EGF), fibroblast growth factor-2 (FGF-2), and brain-derived neurotrophic factor (BDNF), have been reported to promote neural stem cell proliferation and neurogenic activity within adult neurogenic niches [10,11,12,13,14]. Pathological conditions such as ischemia trigger dynamic alterations in the expression of these growth factors, particularly within the subventricular zone and peri-lesional regions, where injury-associated upregulation of EGF, FGF-2, and related trophic signals has been linked to activation of endogenous neural stem and progenitor cells [15].

Previous studies have progressively elucidated the mechanisms underlying neurogenesis. Adult neurogenesis in the subventricular zone (SVZ) and subgranular zone (SGZ) is regulated by multiple, partially overlapping signaling pathways, including EGF/EGF receptor signaling [6,10,16], dopamine–EGF receptor crosstalk [17,18,19,20], Wnt/β catenin signaling [21,22], serotonergic and GABAergic neurotransmission [23,24], BDNF [11], and activity- or stress-related mechanisms [23,25,26]. These pathways collectively govern the activation state, proliferation, and lineage progression of neural stem and progenitor cells within the adult brain [24].

Furthermore, several pharmacological agents have been reported to modulate adult neurogenesis in vivo, and these compounds have also been utilized to investigate the underlying mechanisms of neurogenesis [27,28]. For instance, the neurogenic compound NSI-189 has demonstrated efficacy in preclinical models and has progressed to clinical evaluation [29,30,31]. In parallel, repurposed agents such as metformin and selective serotonin reuptake inhibitors (SSRIs) have been shown to influence adult neurogenesis via metabolic- or neurotransmitter-dependent mechanisms [23,25,26]. Moreover, experimental modulation of dopaminergic signaling and activation of protein kinase C (PKC), using compounds such as prostratin, have been widely utilized as pharmacological tools to stimulate neural stem and progenitor cell proliferation [32]. Given the mechanistic diversity underlying pharmacologically induced neurogenesis, phenotype-based screening approaches are more commonly employed than target-based screening to identify compounds that promote neurogenesis. Moreover, the neurogenic effects elicited by pharmacological agents differ markedly across distinct neurogenic niches and are highly dependent on experimental conditions. Nevertheless, accumulated evidence indicates that the effects of currently available compounds on adult neurogenesis are often modest and variable, underscoring the need to explore alternative strategies capable of more robustly and reliably enhancing adult neurogenesis [31].

Previous studies have performed phenotype-based screening using human fetal hippocampal neural stem/progenitor cells (NSCs/NPCs), such as the HIP-009 cells. In these reports, compound screening was conducted after the HIP-009 cells had been expanded in culture medium containing EGF and FGF to maintain a proliferative state [33]. In contrast, we aimed to establish a more stringent screening paradigm by identifying compounds that support NSC proliferation even in the absence of exogenous growth factors such as EGF and FGF. In this study, we conducted a human neural stem cell–based phenotypic screening under defined, growth factor–free conditions to identify small molecules capable of enhancing neural stem cell proliferation and neurogenic activity.

## 2. Results

### 2.1. Development and Validation of a Phenotypic Screening Platform for NSC Proliferation

**Figure 1:**
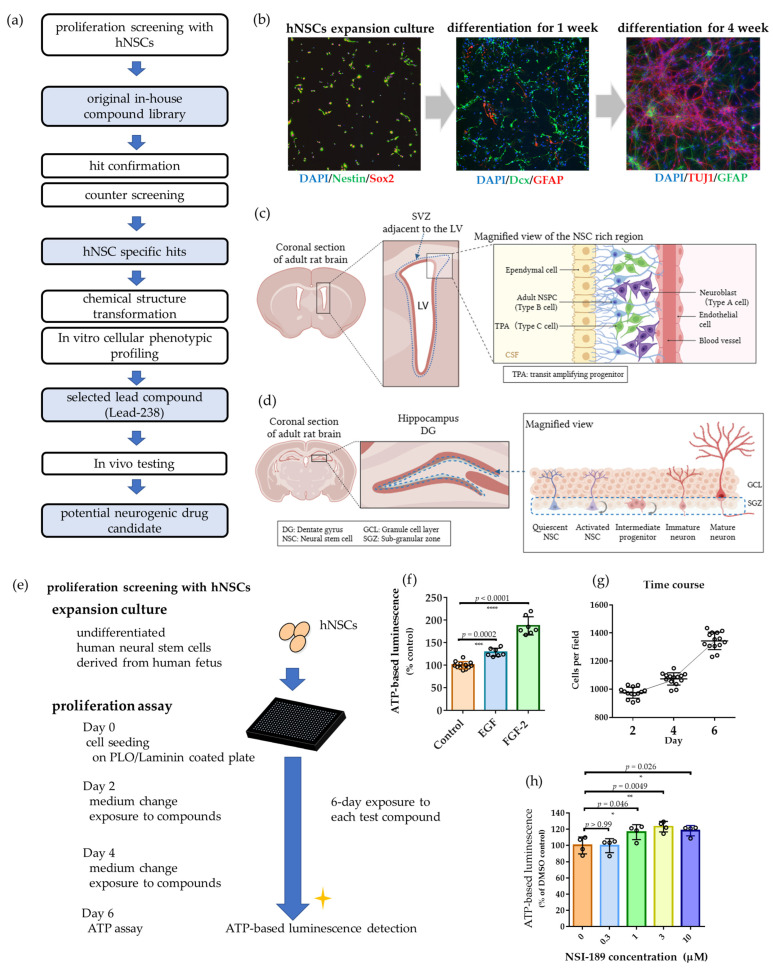
Screening and characterization of human neural stem cells (hNSCs) for neurogenic compound discovery. (**a**) Schematic workflow of the compound screening process using hNSCs. The process includes initial proliferation screening, hit confirmation, counter-screening with a non-neuronal epithelial cell line, identification of hNSC-specific hits, chemical structure optimization, in vitro cellular phenotypic profiling, and in vivo testing of the selected lead compound (Lead-238). (**b**) Immunofluorescence analysis of human neural stem cells and their differentiation. (Left) Undifferentiated human neural stem cells (hNSCs) stained with DAPI (blue), Nestin (green), and SOX2 (red). (Middle) Cells differentiated for 1 week, stained with DAPI (blue), DCX (green), and GFAP (red). (Right) Cells differentiated for 4 weeks, stained with DAPI (blue), TUJ1 (red), and GFAP (green). Images were acquired using a 10x objective lens. (**c**) High-magnification images of the SVZ showing representative cellular composition within the neurogenic niche. (**d**) High-magnification images of the hippocampal dentate gyrus showing the subgranular zone (SGZ). (**e**) Experimental design of the proliferation assay using undifferentiated hNSCs derived from human fetal tissue. Cells were seeded on poly-L-ornithine/laminin-coated plates (Day 0), followed by medium changes and compound exposure on Days 2 and 4. Cell proliferation was assessed on Day 6 using an ATP-based luminescence assay. (**f**) Growth factor (GF) responsiveness of hNSCs. ATP-based proliferation assays were performed under conditions with FGF-2, EGF, or without growth factors. (**g**) Time-course analysis of hNSC proliferation under growth factor-free conditions. Nuclear counts were assessed by DAPI staining on Days 2, 4, and 6, and normalized. The calculated doubling time (Day 2 to Day 6) was approximately 200.4 h. (**h**) Effects of NSI-189 on hNSC proliferation at different concentrations. The x-axis represents compound concentrations, and the y-axis shows luminescence intensity as a percentage relative to the DMSO control (set at 100%). Individual data points are shown together with mean ± SD. Exact *p*-values obtained from Dunnett’s multiple-comparison test are directly annotated on each graph. Statistical significance: * *p* < 0.05, ** *p* < 0.01, *** *p* < 0.001, **** *p* < 0.0001 (Dunnett’s multiple comparisons test).

To identify compounds that promote NSC proliferation, we established a multi-step phenotypic screening cascade using human fetal-derived NSCs. The workflow incorporated an ATP-based primary assay, secondary and counter-screens, structural optimization, in vitro phenotypic profiling, and in vivo validation (Figure 1a). Figure 1a depicts the overall workflow, screening objectives, and assessment parameters at each stage. Primary screening was conducted using an ATP assay [33,34], followed by retesting and counter-screening [35], which enabled the identification of NSC-specific proliferation-promoting compounds. Brain-permeable candidates were selected through structural optimization. Their in vitro activity profiles were evaluated by nuclear count, BrdU incorporation, and the percentages of SOX2-positive and DCX-positive cells. The lead compound was subsequently assayed in vivo to determine its neurogenic potential (Figure 1c,d), completing the screening pipeline from cellular assays to animal-level validation.

Prior to screening, we verified that the human fetal-derived NSCs used in this study retained neurogenic differentiation capacity. Under differentiation conditions, these cells expressed lineage-specific markers, including DCX (immature neurons), TUBB3 (neurons), and GFAP (astrocytes), as shown in Figure 1b, indicating their ability to generate both neuronal and glial lineages. The observed differentiation pattern and marker expression were consistent with previously reported profiles for human fetal NSCs [36,37], supporting the physiological relevance of the model. This validation confirmed the model’s physiological relevance and the suitability of the NSCs for phenotypic screening of NSC proliferation compounds.

Next, as a primary screening step, we evaluated NSC proliferation six days after compound treatment under growth factor–free conditions. Several assays are commonly used as indicators of neural stem cell proliferation, including BrdU incorporation, tetrazolium-based assays such as WST-8 or MTT, and ATP-based assays, and the correlations among these assays have been previously reported [38]. Among them, ATP-based assays exhibit high sensitivity and are widely employed in high-throughput screening. Therefore, we adopted an ATP-based assay to assess NSC proliferation in our screening platform (Figure 1e). Undifferentiated human fetal NSCs were expanded and seeded on poly-L-ornithine/laminin-coated plates (Day 0), with compounds added at seeding. Media containing compounds were refreshed on Days 2 and 4, and ATP levels were quantified on Day 6 using a luminescence-based assay. This assay design ensured consistent compound exposure and robust measurement of proliferative responses.

Proliferation conditions were further evaluated by comparing growth factor-supplemented and growth factor-free media (Figure 1f). FGF-2 supplementation resulted in a 1.87-fold increase in ATP levels, indicating enhanced proliferation, whereas EGF, despite producing a statistically significant increase, exerted only a modest effect. These findings suggest that the NSCs exhibit low responsiveness to EGF under basal conditions, a property typically associated with quiescent SVZ stem cells [10,17,20].

Under growth factor-free conditions, NSCs maintained basal proliferative activity, as DAPI staining revealed an approximately 1.2-fold increase in nuclear count over two-day intervals (Day 2 to Day 4, and Day 4 to Day 6; Figure 1g). Based on these data, the calculated doubling time was approximately 200.4 h, which is markedly slower than previously reported values under growth factor-supplemented conditions [38]. These results confirmed that NSCs retained basal proliferation under growth factor-free conditions, enabling quantitative assessment of compound-induced proliferation-promoting effects.

In this condition, NSI-189, previously reported to enhance NSC proliferation, induced statistically significant but relatively modest increases in ATP levels across the tested concentration range (Figure 1h). Similarly, other known modulators, including the p38 MAPK inhibitor SB239063, the cdc25 phosphatase inhibitor NSC95397, and the ROCK inhibitor Y-27632, exhibited weak but detectable proliferative effects under growth factor-free conditions (Figure S1), consistent with previous reports [39]. These findings confirm that the assay reliably detects known neurogenic compounds, supporting its validity for identifying novel agents with stronger activity.

To validate the screening system using a 384-well plate format, the Z′-factor was calculated based on luminescence values from positive (FGF-2) and negative (DMSO) controls, following standard methodology [40]. The calculated Z′-factor was 0.826, which exceeds the threshold for excellent assay quality (>0.8), confirming the robustness of the screening system (Figure S2).

### 2.2. Library Screening and Identification of Neurogenic Hit Compounds

We first constructed an in-house compound library to maximize structural diversity and drug-like properties for the identification of novel neurogenic agents. To distinguish our screening set from previously evaluated pathway-focused libraries [37], we adopted a design principle emphasizing a high fraction of sp^3^-hybridized carbons (Fsp^3^) and selected compounds that complied with Lipinski’s Rule of Five, resulting in a collection of 178 compounds [41,42,43].

**Figure 2:**
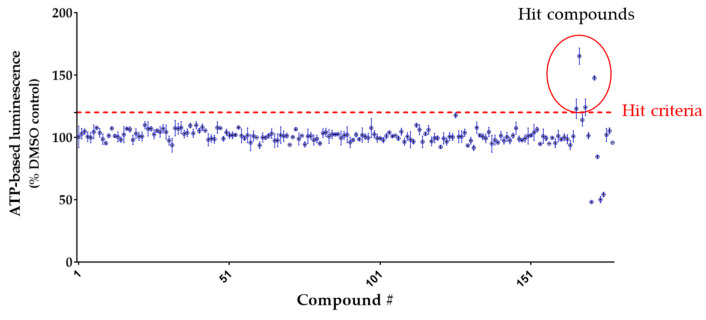
Phenotypic screening for compound library using hNSCs. Screening results from the lead compound library. Compounds were tested at 1 μM. The x-axis represents compound identifiers, and the y-axis shows luminescence intensity as a percentage relative to the DMSO control (set at 100%). Hit compounds were defined as those exceeding the threshold of mean + 4 × SD of DMSO control (red dotted line). Data are mean ± SD, *n* = 4.

Following library design, phenotypic screening was performed under growth factor-free conditions to identify compounds promoting NSC proliferation. Cell proliferation was assessed by ATP-based luminescence measurements after 6 days of compound exposure at 1 μM concentration. Four newly identified compounds (HIT-695, HIT-696, HIT-698, and HIT-701) exhibited ATP levels exceeding 122.4% of the DMSO control (mean + 4 × SD, *n* = 4; Figure 2) and were identified as hit compounds. These hits showed consistent activity across replicates and were selected for further evaluation based on their robust proliferative effects. The ATP levels for these compounds were 123.0 ± 7.8%, 165.1 ± 6.6%, 124.1 ± 6.7%, and 147.6 ± 1.8%, respectively (*n* = 4).

### 2.3. Concentration-Response Characterization of Hit Compounds

**Figure 3:**
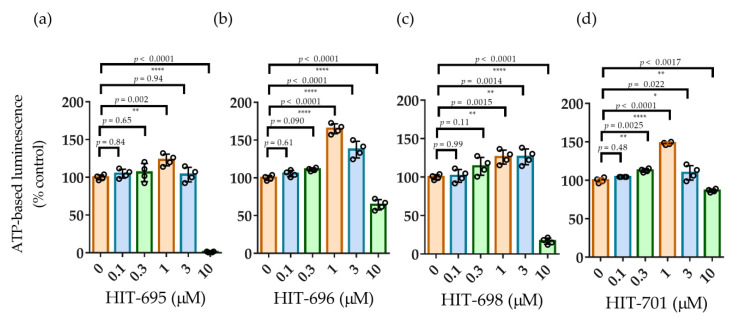
Concentration-response analysis of hit compounds in hNSCs. (**a**–**d**) Evaluation of concentration-response activities of HIT-695, HIT-696, HIT-698, and HIT-701. The x-axis shows compound concentrations, and the y-axis shows luminescence intensity relative to DMSO control (100%). Individual data points are shown together with mean ± SD, *n* = 4. Exact *p*-values obtained from Dunnett’s multiple-comparison test are directly annotated on each graph. Statistical significance: * *p* < 0.05, ** *p* < 0.01, **** *p* < 0.0001 (Dunnett’s multiple comparisons test).

Reproducibility and selectivity of the hit compounds were assessed through ATP-based concentration-response analysis and counter-screening in a non-neural cell line. In the concentration-response assay, all four hit compounds (HIT-695, HIT-696, HIT-698, HIT-701) consistently increased ATP levels in hNSCs in a concentration-dependent manner, reaching maximal activity at 1 µM (123–165%) with reduced activity at ≥3 µM (Figure 3a–d). Notably, three of the compounds (HIT-695, HIT-696, and HIT-698) reduced ATP levels to below control (DMSO) values at 10 μM. Taken together, these findings indicate that the compounds sharing this common core scaffold exhibit NSC proliferative activity. To confirm cellular selectivity, the proliferative effects of the hit compounds were evaluated in HT-29 cells (Figure S3). Under these conditions, no significant elevation in ATP levels was detected, supporting the neural selectivity of the hit compounds.

### 2.4. Selection of Lead-238 and Evaluation of Its In Vitro Cellular Profiling

To eliminate cytotoxicity at higher concentrations while maintaining the chemotype associated with NSC-selective activity and improving the ADME properties, a series of structural optimization steps was undertaken. Through this process, we ultimately identified Lead-238, an optimized candidate featuring an all-sp^3^ bicyclic core framework consistent with a bicyclo[3.3.0] topology. Key calculated physicochemical properties of Lead-238 are summarized in Table S1. Lead-238 was subsequently evaluated using in vitro dose–response and phenotypic assays to characterize its proliferative and neurogenic effects in human neural stem cells.

**Figure 4:**
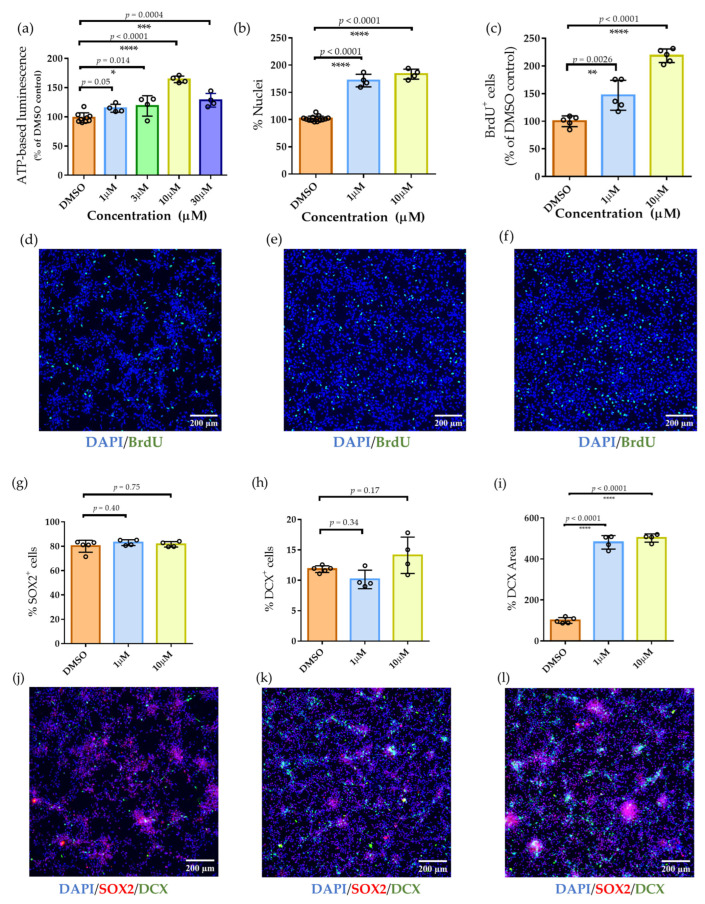
Effects of Lead-238 on proliferation and phenotypic profiling of human neural stem cells (hNSCs). (**a**) ATP-based luminescence assay showing dose-dependent increases in cellular ATP levels following treatment with Lead-238 at 1, 3, 10, and 30 μM. Individual data points are shown together with mean ± SD (DMSO: *n* = 9; all treatment concentrations: *n* = 4 each). (**b**) Quantification of total nuclei count (% of DMSO control) after treatment with 1 and 10 μM Lead-238. Individual data points are shown together with mean ± SD (DMSO: *n* = 13; all treatment concentrations: *n* = 4 each). (**c**) Percentage of BrdU-positive cells (% of DMSO control) indicating proliferative activity under the same treatment conditions. Individual data points are shown together with mean ± SD (*n* = 5 each). (**d**–**f**) Representative immunofluorescence images of hNSCs stained with DAPI (blue, nuclei) and BrdU (green, proliferating cells) following treatment with (**d**) DMSO, (**e**) 1 μM Lead-238, and (**f**) 10 μM Lead-238. (**g**) Percentage of SOX2-positive cells after treatment with DMSO, 1 μM, and 10 μM Lead-238. Individual data points are shown together with mean ± SD (DMSO: *n* = 5; treatment groups: *n* = 4 each) (**h**) Percentage of DCX-positive cells under the same conditions. Individual data points are shown together with mean ± SD (DMSO: *n* = 5; treatment groups: *n* = 4 each). (**i**) Quantification of DCX-positive area (%), showing significant expansion at 1 and 10 μM. Individual data points are shown together with mean ± SD (DMSO: *n* = 5; treatment groups: *n* = 4 each). (**j**–**l**) Representative immunofluorescence images of hNSCs stained with DAPI (blue), SOX2 (red, stem cell marker), and DCX (green, immature neuron marker) following treatment with (**j**) DMSO, (**k**) 1 μM Lead-238, and (**l**) 10 μM Lead-238. Scale bars: 200 μm. Exact *p*-values obtained from Dunnett’s multiple-comparison test are directly annotated on each graph. Statistical significance: * *p* < 0.05, ** *p* < 0.01, *** *p* < 0.001, **** *p* < 0.0001 (Dunnett’s multiple comparisons test).

The in vitro activity of Lead-238 was confirmed using concentration-dependent ATP assays, which demonstrated significant proliferative effects in NSCs. Under growth factor-free conditions, Lead-238 increased ATP levels in a dose-dependent manner, reaching 115.1 ± 4.8% at 1 μM and 165.5 ± 3.5% at 10 μM, relative to the DMSO control (DMSO: *n* = 9; all treatment concentrations: *n* = 4 each; Figure 4a). Using the high-sensitivity ATP assay, we determined the EC_50_ value for the NSC-proliferation–enhancing effect of Lead-238 by applying a four-parameter logistic (4PL) model. Because a decrease in ATP levels was observed at the 30 μM concentration, the maximal response (E_max) was fixed at the value obtained at 10 μM, which produced the most reproducible proliferative effect. Based on the fitted curve, the EC_50_ for Lead-238 was calculated to be 4.1 μM. The results were generally consistent with those obtained from BrdU incorporation, nuclear counts, and DCX-positive area measurements.

Lead-238 treatment significantly increased NSC proliferation, as demonstrated by elevated total cell counts and enhanced BrdU incorporation (Figure 4b–f). Quantitative analysis of DAPI-stained nuclei revealed a dose-dependent increase in cell number, with 1 µM and 10 µM treatments resulting in 1.64 ± 0.11-fold and 1.83 ± 0.07-fold increases, respectively, compared to control (DMSO: *n* = 13; all treatment concentrations: *n* = 4 each). BrdU incorporation assays showed a statistically significant increase in the proportion of BrdU-positive cells at both concentrations (1 µM: *p* = 0.0026; 10 µM: *p* < 0.0001; *n* = 5), with BrdU incorporation rates of 1.47 ± 0.27-fold and 2.18 ± 0.13-fold of control for 1 µM and 10 µM, respectively. These findings are consistent with ATP assay results, further validating the proliferative effect of Lead-238.

In addition to promoting proliferation, Lead-238 enhanced neurogenic output without altering lineage balance. The area of DCX-positive regions increased significantly at both 1 µM and 10 µM (*p* < 0.001; Figure 4l), with fold changes of 4.81 ± 0.32 and 5.02 ± 0.42, respectively (*n* = 4). The proportions of SOX2- and DCX-positive cells remained unchanged across treatment conditions (Figure 4g–h), indicating that while the absolute numbers of these populations increased, their relative abundance was maintained. These results suggest that Lead-238 promotes expansion of immature neurons while preserving stem cell marker expression.

### 2.5. Mechanism Analysis

To further investigate the mechanism underlying NSC proliferation, we evaluated whether the observed effects of Lead-238 were mediated through signaling pathways previously implicated in neurogenic activity, specifically PKC activation and dopamine receptor signaling.

**Figure 5:**
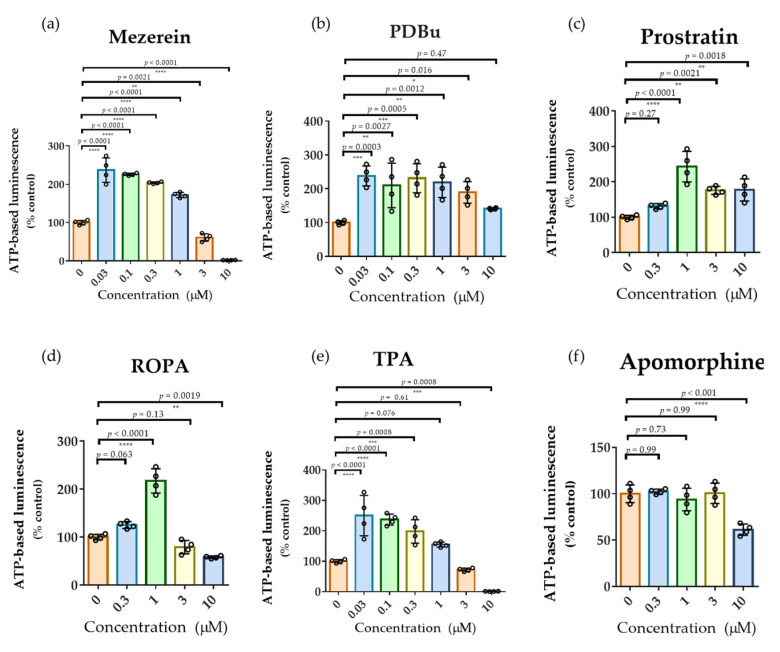
Mechanistic evaluation of PKC and GPCR pathways in the hNSCs. (**a**–**f**) Effects of various compounds on hNSC proliferation at different concentrations. The x-axis represents compound concentrations, and the y-axis shows luminescence intensity as a percentage relative to the DMSO control (set at 100%). Compounds tested include: (**a**) Mezerein, (**b**) PDBu, (**c**) Prostratin, (**d**) ROPA, (**e**) TPA, (**f**) Apomorphine. Individual data points are shown together with mean ± SD (*n* = 4). Exact *p*-values obtained from Dunnett’s multiple-comparison test are directly annotated on each graph. Data are presented as mean ± SD, *n* = 4. Statistical significance: * *p* < 0.05, ** *p* < 0.01, *** *p* < 0.001, **** *p* < 0.0001 (Dunnett’s multiple comparisons test).

Representative PKC activators (Mezerein (toxic diterpene ester), PDBu (strong phorbol ester), ROPA (mild phorbol ester), TPA (toxic phorbol ester), Prostratin (non-tumor phorbol ester)) were tested under the same assay condition used for phenotypic screening [29,32,39]. These compounds induced robust increases in ATP levels, reaching up to 250% of control values (Figure 5a–e), demonstrating that the assay is responsive to PKC-mediated proliferation, consistent with previous reports [32]. In addition, cytotoxic effects of PKC activators, such as Mezerein and TPA, were observed at these higher concentrations. In contrast, in silico docking simulations against multiple PKC isoforms [44,45], including PKCα, PKCβ, PKCδ, and PKCε, indicated that Lead-238 did not exhibit significant binding to PKC C1A or C1B domains (Figure S5). Consistent with these findings, an in vitro binding assay using the PKCα C1B domain revealed no detectable interaction with Lead-238 (Table S2; based on the methodological approach in ref. [46]). These results suggest that the proliferative effect of Lead-238 is not mediated through a direct action on PKC activation.

To assess the involvement of GPCR, the dopamine agonist apomorphine was evaluated under the same conditions. Apomorphine did not induce significant changes in ATP levels (Figure 5f), suggesting that dopaminergic signaling does not strongly contribute to NSC proliferation in this assay context. Furthermore, GPCR functional profiling demonstrated that Lead-238 did not activate dopamine D1–D5 or 5-HT_2_ receptors (Table S3; based on the methodological approach described in ref. [47]). These results suggest that the proliferative effect of Lead-238 is not mediated through direct actions on dopamine or serotonin receptors.

### 2.6. Pharmacokinetic Evaluation to Support In Vivo Dosing

To inform the in vivo study design, pharmacokinetic characterization of Lead-238 was performed in male SD rats (Table 1, Figure S4). In the initial experiment, animals received a single oral dose of 2 mg/kg, and brain and plasma samples were collected 1 h post-administration to determine the brain-to-plasma partition coefficient (K_p_), which was calculated to be 18.3. In a subsequent study, rats were administered a single oral dose of 20 mg/kg, and plasma samples were collected at multiple time points (0.25–24 h) to quantify drug concentrations in plasma. Brain concentrations at each time point were estimated using plasma levels and the previously determined K_p_ value. Based on the resulting exposure profiles, a dose of 30 mg/kg was predicted to maintain brain concentrations above 1 µM for approximately 4 h (from 0.5 to 4 h post-dose).

Collectively, these results indicate that oral administration of Lead-238 can achieve sustained brain exposure at levels predicted to elicit pharmacological effects in vivo.

### 2.7. In Vivo Evaluation of Neurogenic Effects of Lead-238

**Figure 6:**
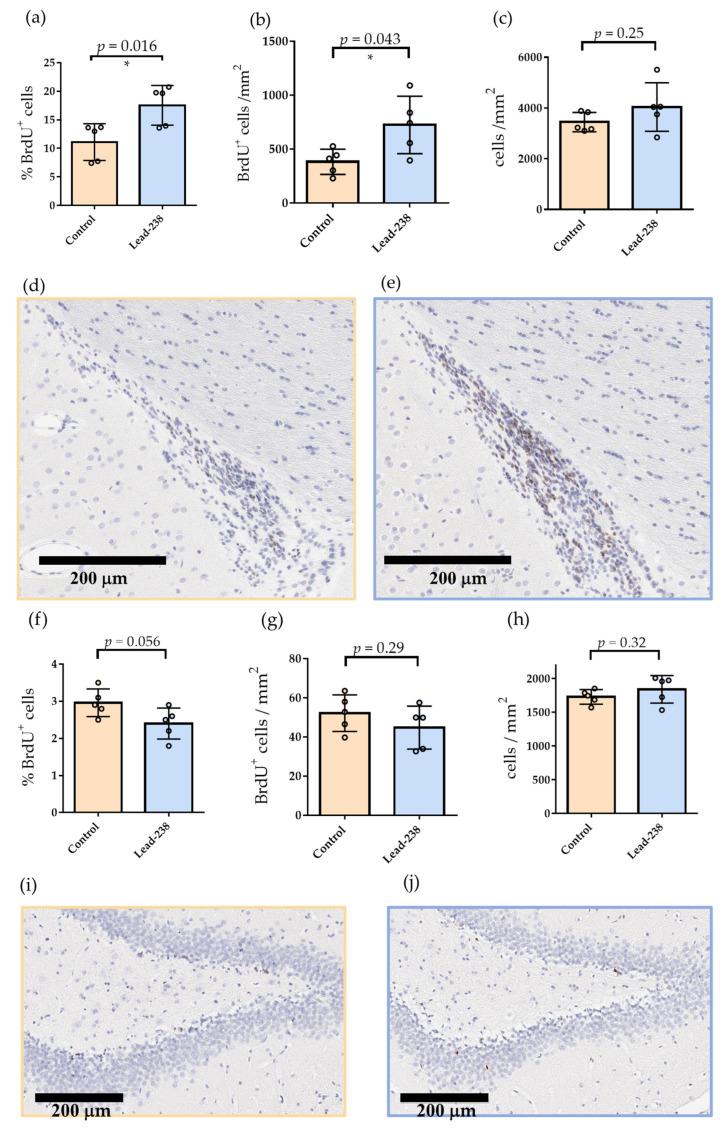
Quantitative analysis of Lead-238-induced cell proliferation in the SVZ and SGZ. Male Wistar rats (8 weeks old) were administered Lead-238 (30 mg/kg, orally) and BrdU (50 mg/kg, intraperitoneally) twice daily for 6 consecutive days. (**a**–**e**) Quantification of BrdU-positive cells in the subventricular zone (SVZ): (**a**) Percentage of BrdU-positive cells, (**b**) Absolute number of BrdU-positive cells per mm^2^, (**c**) Total number of cells per mm^2^. (**d**,**e**) Representative images of SVZ sections in control (**d**) and Lead-238-treated (**e**) animals, stained for BrdU using DAB (brown) and counterstained with hematoxylin (blue). Total cell counts were determined based on hematoxylin-stained nuclei. (**f**–**j**) Quantification of BrdU-positive cells in the subgranular zone (SGZ): (**f**) Percentage of BrdU-positive cells, (**g**) Absolute number of BrdU-positive cells per mm^2^, (**h**) Total number of cells per mm^2^. (**i**,**j**) Representative images of SGZ sections in control (**i**) and Lead-238-treated (**j**) animals, stained for BrdU using DAB (brown) and counterstained with hematoxylin (blue). Scale bars: 200 μm. Individual data points are shown together with mean ± SD (*n* = 5). Exact *p*-values obtained from Student’s *t*-test are directly annotated on each graph. Statistical significance: * *p* < 0.05.

**Figure 7:**
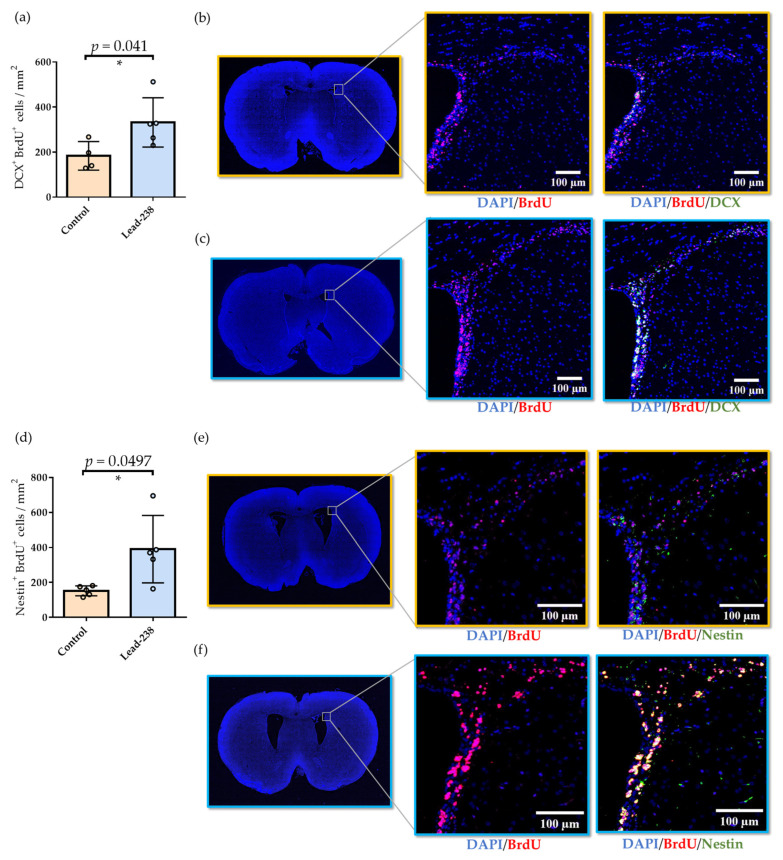
Quantitative analysis of BrdU/DCX- and BrdU/Nestin-positive cells in the SVZ. (**a**) Quantification of BrdU/DCX double-positive cells per SVZ section in control and Lead-238-treated rats. Lead-238 significantly increased the number of BrdU/DCX double-positive cells. (**b**,**c**) Representative fluorescence microscopy images of SVZ regions in control (**b**) and Lead-238-treated (**c**) animals, stained with DAPI (blue), BrdU (red), and DCX (green). Left panels show low-magnification images of whole sections; right panels show magnified views of boxed regions. (**d**) Quantification of BrdU/Nestin double-positive cells per SVZ section in control and Lead-238-treated rats. Lead-238 significantly increased the number of BrdU/Nestin double-positive cells. (**e**,**f**) Representative fluorescence microscopy images of SVZ regions in control (**e**) and Lead-238-treated (**f**) animals, stained with DAPI (blue), BrdU (red), and Nestin (green). Left panels show low-magnification images; right panels show magnified views of boxed regions. Scale bars: 100 μm. Individual data points are shown together with mean ± SD (*n* = 4–5). Exact *p*-values obtained from Student’s *t*-test are directly annotated on each graph. Statistical significance: * *p* < 0.05.

To evaluate the neurogenic effects of Lead-238 in vivo, adult Wistar rats (8 weeks old) were administered Lead-238 (30 mg/kg, orally, twice daily) for six consecutive days, together with BrdU (50 mg/kg, intraperitoneally) to label proliferating cells. Following perfusion fixation, coronal sections containing the SVZ and SGZ were prepared, and BrdU incorporation was visualized by DAB staining in the SVZ (Figure 6a–e) and SGZ (Figure 6f–j). To characterize the phenotypes of proliferating cells, double immunofluorescence for BrdU/DCX or BrdU/Nestin was performed (Figure 7). The distribution and abundance of BrdU-positive cell counts and DCX-positive immature neurons in the SVZ were consistent with previous reports [32,48,49], supporting the validity of the evaluation system.

Lead-238 treatment significantly increased the number of BrdU-positive cells in the SVZ, whereas no significant effect was observed in the SGZ (Figure 6). Compared with controls, the proportion of BrdU-positive cells in the SVZ increased from 11.1 ± 3.2 to 17.54 ± 3.5 (*p* < 0.01), and the absolute number of BrdU-positive cells was also significantly elevated (Control: 382.6 ± 117.4 cells/mm^2^; Lead-238: 724.7 ± 266.5 cells/mm^2^; *p* < 0.05; *n* = 5, Figure 6a–b). In contrast, total SVZ cellularity remained unchanged (Figure 6c), indicating that the increase was specific to proliferating NSCs and neuroblasts. Representative images of the SVZ are shown in Figure 6d,e. In the SGZ, Lead-238 treatment did not produce a significant change in the number of BrdU-positive cells (Figure 6f–h). Quantitative analysis confirmed the absence of statistically significant differences in BrdU incorporation between control and treated groups (*p* > 0.05). Representative images of the SGZ are shown in Figure 6i,j.

Further analysis of proliferating cells revealed that Lead-238 treatment significantly increased the number of cells co-expressing BrdU and DCX, as well as BrdU and Nestin, in the SVZ, indicating expansion of both neural stem/progenitor cells and immature neurons. Representative whole-coronal section images and high-magnification views of the SVZ are shown in Figure 7, whereas low-magnification overview images of the SVZ are provided in Figure S6. Quantitative analysis showed that the number of BrdU/DCX double-positive cells increased from 183.1 to 331.7 (cells per mm^2^, *n* = 4–5, Figure 7a), while BrdU/Nestin double-positive cells increased from 152.3 to 390.0 (cells per mm^2^, *n* = 5, Figure 7d). Representative images of BrdU-positive cells and BrdU/DCX double-positive cells in control and Lead-238-treated animals are shown in Figure 7b (control) and Figure 7c (Lead-238), respectively. Similarly, representative images of BrdU-positive cells and BrdU/Nestin double-positive cells are shown in Figure 7e (control) and Figure 7f (Lead-238).

## 3. Discussion

Adult neurogenesis in the mammalian brain has been shown to serve distinct functional roles depending on the neurogenic niche. In the subventricular zone (SVZ), adult neurogenesis contributes to functional recovery after brain injury [50,51]. In contrast, neurogenesis in the hippocampal subgranular zone (SGZ) is implicated in learning and memory as well as antidepressant effects. In the intact adult brain, multiple growth factors, including epidermal growth factor (EGF), fibroblast growth factor-2 (FGF-2), and brain-derived neurotrophic factor (BDNF), are known to promote neurogenic activity. Accordingly, expansion of the neural stem cell (NSC) pool within these regions is considered a promising strategy for neural regeneration. Several pharmacological agents capable of enhancing neurogenesis have been reported [19,29,30,31]. However, the therapeutic benefits of these agents are often limited by insufficient efficacy, safety liabilities, or inadequate brain penetration. To address these challenges and facilitate the discovery of novel neurogenic compounds, we established an in vitro evaluation system to assess drug-induced neurogenic activity in the absence of exogenous growth factors, together with the construction of a small-molecule library for favorable ADMET properties. Specifically, to assess neurogenic effects driven solely by compound activity, we developed an in vitro assay system using human NSCs under growth factor-free conditions (Figure 1). Given that previous studies have reported that small molecules identified through screening using HIP-009 cells were able to enhance neural stem cell proliferation in vivo in rats, this screening platform is considered to possess a certain degree of translational relevance [29,30,31,33]. Based on this rationale, we next established an in vivo evaluation platform using rats to assess the biological efficacy of Lead-238 (Figure 1c,d). Using this integrated approach, we screened our proprietary lead library and identified Lead-238, a novel compound capable of expanding the NSC pool in the SVZ.

We further developed a concept-driven phenotypic screening platform that prioritizes NSC proliferation (Figure 1e) and leverages rational chemical library design, enabling the discovery of neurogenic compounds (Figure 2). In contrast to multiparametric platforms that assess cell fate and differentiation [37], our assay selectivity focuses on NSC proliferation. It achieves high robustness (Z′-factor > 0.8) (Figure S2) under conditions that partially approximate the slow proliferative state characteristic of adult neurogenic niches (Figure 1g) [11,12]. To complement this assay, we curated a library of 178 compounds enriched for sp3-rich, three-dimensional scaffolds within drug-like, ADME-compliant chemical space, representing a deliberate departure from conventional mechanism-of-action-focused collections. Saturated, non-planar architectures are increasingly associated with unique biological profiles and improved physicochemical properties [41,42]. This design strategy, combined with physiologically relevant assay conditions, yielded a comparatively high hit rate (2.2%), underscoring a productive alignment between chemical space and biological response. Notably, all four hits (Figure 3) converged on a common chemotype that is structurally distinct from previously reported neurogenic compounds, highlighting a novel scaffold with potential biological relevance. By integrating structure-response relationships across active and inactive members of this series, we selected Lead-238 as a brain-penetrant compound (Table 1 and Table S1, Figure S4) exhibiting robust neurogenic activity (Figure 4a). Collectively, these findings demonstrate that a proliferation-centric phenotypic screening strategy, combined with rational 3D library design, can accelerate the discovery of translatable neurogenic compounds.

Lead-238 exhibited sustained neurogenic activity across a broad concentration range (Figure 4a–f), in sharp contrast to NSI-189, which elicited only modest effects (Figure 1h), consistent with its lack of clinical efficacy [31]. In contrast to mTOR/PI3K/MEK inhibitors or erythropoietin, which have been reported to disrupt lineage balance [37] or deplete the NSC pool [52,53], Lead-238 enhances neurogenic activity while preserving physiological lineage proportions (Figure 4). This activity was accompanied by increased BrdU incorporation and expansion of DCX-positive areas, without altering the relative proportions of SOX2 and DCX-positive cells, indicating maintenance of lineage balance. Neural selectivity was further supported by the absence of proliferative effects in non-neural cells (Figure S4). Unlike agents with limited central nervous system (CNS) exposure, such as erythropoietin and prostratin, Lead-238 combined robust neurogenic activity with effective brain penetration (Table 1), thereby reinforcing its translational potential. Collectively, these features position Lead-238 as a distinctive and promising agent for CNS regeneration.

Lead-238 exhibits a distinctive ability to selectively enhance neurogenesis in the SVZ. However, the mechanisms underlying it remain unclear. As shown in Figure 5a–e, multiple PKC activators significantly increased ATP levels in our in vitro assay system. Based on this observation, we investigated whether Lead-238 interacts with the PKC signaling pathway. Specifically, the potential interaction of Lead-238 with PKCα was evaluated using both in silico simulations and in vitro binding assays (Table S2, Figure S5). In contrast, its interactions with other PKC isoform domains were assessed exclusively through in silico analyses (Figure S5).

Contrary to our initial expectations, Lead-238 selectively enhanced neurogenic activity in the SVZ in vivo with no detectable effects in the hippocampal SGZ (Figure 6 and Figure 7). In contrast, NSI-189, which we evaluated using our in vitro assay (Figure 1), has been shown in rodent in vivo models to predominantly enhance neurogenesis in the subgranular zone (SGZ), while exerting minimal effects on the subventricular zone (SVZ) [19,20,21]. These findings suggest that while in vitro assays have limitations in distinguishing SVZ- versus SGZ-specific effects, they are still useful for identifying compounds that enhance neurogenesis in either or both regions. In addition, several neurogenic compounds have been reported to exhibit niche-specific activities. SSRIs (e.g., fluoxetine) reliably enhance SGZ proliferation in vivo and show no SVZ activation [23]. Metformin mainly promotes SGZ neurogenesis in vivo, with occasional SVZ effects under specific injury-related conditions [25,26]. Dopamine agonists can stimulate SVZ proliferation in vivo [17,18], but do not induce detectable responses under our growth factor–free in vitro conditions (Figure 5f). Indeed, GPCR functional profiling demonstrated that Lead-238 did not activate dopamine D1-D5 or 5-HT_2_ receptors (Table S3). In addition, BIO (a canonical Wnt pathway activator) failed to enhance NSC proliferation under our growth factor–free assay conditions, despite showing clear activity in the presence of exogenous growth factors (Figure S1). Therefore, these observations suggest that Wnt/β-catenin signaling, as well as downstream pathways such as ERK and AKT, are unlikely to play a major role in mediating the proliferative effects of Lead-238 under these assay conditions. These findings suggest that our growth factor–free assay might better reflect a stem-like, slowly proliferating NSC state (Figure 1g) than predominantly capture transit-amplifying progenitors (TAPs). Given that classical SVZ mitogens such as EGF, dopamine agonists, and canonical Wnt activators typically target rapidly dividing TAPs, their limited activity in our assay may simply reflect the low-proliferative cellular state of the fetal NSCs examined. From this perspective, the activity of Lead-238 may indicate that it affects a more primitive NSC population upstream of TAPs, which could help explain its SVZ-selective neurogenic effects observed in vivo. Taken together, these results strongly suggest that the neurogenic effects of Lead-238 are not mediated, at least in part, through the PKC, dopamine serotonin, or canonical Wnt signaling pathways. These distinct regional profiles reflect the fundamentally different regulatory landscapes governing the two neurogenic niches [24].

SVZ neurogenesis is shaped by the broad integration of extrinsic cues, including EGF/EGFR signaling [6,10,16], Wnt/β-catenin pathways [21,22], dopamine–EGFR crosstalk [17,18,19,20], Notch-dependent maintenance signals [54], Sonic hedgehog [55], BMP4/7 antagonism (e.g., Noggin) [56], and neurotransmitter inputs such as GABA, acetylcholine, glutamate, and serotonergic axons [57]. In contrast, SGZ neurogenesis relies more heavily on serotonergic input, tonic GABAergic inhibition [23,24], BDNF–TrkB signaling [11], cholinergic [58] and noradrenergic modulation [59], and environmental or metabolic cues such as exercise-induced VEGF/BDNF [60] and stress-associated glucocorticoids [61,62]. Given that our growth factor–free fetal NSC assay does not fully recapitulate the cellular diversity and circuit-level inputs present in the adult SGZ niche, it may have limited sensitivity to mechanisms that depend on complex in vivo interactions. Accordingly, conclusions based solely on in vitro signaling events should be interpreted with caution, and future studies incorporating in vivo cellular connectivity and niche-level interactions will be necessary to fully delineate the underlying mechanisms. Although we initially assumed that our screening platform would possess a certain degree of clinical relevance based on prior studies, the selective activity of Lead-238 toward the SVZ rather than the SGZ indicates that its translational applicability may be only partial. In vivo, SGZ neurogenesis is strongly shaped by interactions with local niche components—including glial cells, extracellular matrix signals, and afferent circuit activity—that are absent from our simplified in vitro system. As a result, the assay may preferentially detect SVZ-like proliferative responses rather than SGZ-specific regulatory mechanisms. Under pathological conditions such as stroke, SGZ neurogenesis can be aberrantly enhanced by inflammation-linked cytokine signaling and disrupted GABAergic tone, promoting maladaptive circuit remodeling, cognitive decline, and seizure susceptibility [63,64,65], whereas SVZ-derived neurogenesis more often contributes to reparative migration toward injured areas. In this context, the ability of Lead 238 to selectively expand BrdU^+^/Nestin^+^ and BrdU^+^/DCX^+^ SVZ progenitors—despite the minimal responsiveness of our assay to classical SVZ mitogens such as EGF, dopamine, or Wnt activators (Figure 5f and Figure S1)—suggests that Lead-238 may act through previously unrecognized upstream mechanisms that preferentially activate the SVZ niche, although further studies will be required to clarify the molecular pathways involved.

Identifying the molecular target underlying SVZ selectivity of Lead-238 represents a critical next step to determine whether its effects are mediated through a previously unrecognized mechanism and to enable rational optimization of this compound. Understanding whether SVZ neurogenesis promoted by Lead-238 translates into changes in physiological functions, including cognitive abilities, will be an important step toward defining the functional relevance of SVZ activation. Together with these assessments, elucidating the mechanisms by which Lead-238 activates NSCs within the SVZ, together with evaluating its efficacy across diverse disease models, has the potential to provide new insights into regenerative medicine strategies. Given the established role of SVZ-derived progenitors in injury-induced repair, these findings may also imply potential relevance in contexts such as post-stroke recovery or other injury-associated conditions.

At the same time, several limitations of the present study should be acknowledged. First, the chemical structure of Lead-238 remains undisclosed due to proprietary restrictions. Second, the in vitro assays utilized human fetal NSCs rather than adult-derived NSCs, which may differ in proliferative dynamics and niche responsiveness. Third, no functional or behavioral assessments were performed, and therefore, the physiological relevance of enhanced SVZ neurogenesis remains to be determined. Finally, the in vivo evaluation relied on a single-dose paradigm, and future studies incorporating repeated-dose regimens and broader PK/PD characterization will be required to define the translational potential of Lead-238 more fully.

## 4. Materials and Methods

### 4.1. Human Neural Stem Culture for Expansion and Differentiation

HIP-009 cells were obtained from PhoenixSongs Biologicals, Inc. (Branford, CT, USA, Cat#23002-009). Cells were expanded according to the manufacturer’s instructions. Culture dishes were coated with 10 μg/mL laminin (Corning, Corning, NY, USA, Cat#354232) in PBS (Nacalai Tesque, Inc., Kyoto, Japan, Cat#14249-24). Cells were seeded and grown on laminin-coated dishes in NeuralStemCell Growth Medium (PhoenixSongs Biologicals, Cat#21001-250), or RHB-A medium (Takara Bio, Shiga, Japan, Cat#Y40001) supplemented with 10 ng/mL FGF-2 (bFGF; PeproTech, Rocky Hill, NJ, USA, Cat#100-18B), and 20 ng/mL epidermal growth factor (EGF; PeproTech, Cat#AF-100-15). The medium was changed every 2–3 days, and confluent cells were split for expansion every 4–5 days. Cells were incubated at 37 °C in a humidified atmosphere of 5% CO_2_. Before the start of differentiation, expanded cells were plated on laminin-coated plates in Neural Transition Medium (PhoenixSongs Biologicals, Cat#21003-250) and cultured for 3 days. Next, cells were seeded on poly-D-lysine (PDL: Sigma-Aldrich, Burlington, MA, USA, Cat#P7280)-coated plates in Neural Differentiation medium (PhoenixSongs Biologicals, Cat#21004-250) for differentiation. The differentiation process was performed for 28 days, during which half of the medium was changed twice a week. During differentiation, cell culture was performed at 37 °C in a humidified atmosphere of 2% O_2_ and 6% CO_2_. HIP-009 cells at passages 8 to 12 were used. This study was approved by the Sumitomo Pharma Research Ethics Committee.

### 4.2. Chemical Screening

The chemical screening system was developed by modifying and optimizing a previously reported protocol [66]. 384-well plates (Aurora Microplates, Scottsdale, AZ, USA, Cat #ABM2-11101A) were coated with poly-L-ornithine (PLO; Sigma-Aldrich, Cat#P3655) and 10 μg/mL laminin sequentially. Expanded HIP-009 cells were seeded in the coated plates at a density of 3500 cells per well in N-2-supplemented DMEM/F12 (GIBCO, Waltham, MA, Cat#12400-024) containing 0.1% B27 supplement (GIBCO, Cat#17504044) using Micro Shot 705 (MS TECHNOS Co., Ltd., Tokyo, Japan). Each compound was dissolved in medium, and the solution was added to each well of the assay plate at a final concentration of 1 μM using EDR-384SR (BIOTEC Co., Ltd., Tokyo, Japan). The cells were then incubated at 37 °C in a humidified atmosphere of 5% CO_2_. The endpoint was set at Day 6, and the medium containing the test compound was replaced every 48 h using EDR-384SR. Cell proliferative activity was evaluated using CellTiter-Glo (Promega, Madison, WI, USA, Cat#G7570) according to the manufacturer’s protocol. After cells were subjected to CellTiter-Glo, luminescence was measured with a plate reader (Envision, Revity, Waltham, MA, USA). We constructed a library of 178 signal transduction compounds. Screening data visualization was performed using TIBCO Spotfire v5.0 (Cloud Software Group, Inc., Fort Lauderdale, FL, USA).

### 4.3. Immunostaining and Fluorescence Analysis

Cells were fixed with 4% paraformaldehyde (FUJIFILM Wako Pure Chemical Corporation, Osaka, Japan, Cat#163-20145) for 20 min at room temperature (RT). For the BrdU assay, N2 medium containing BrdU (100 μM; Sigma-Aldrich, Cat##B5002) was added 1 h before fixation. After 2–3 rinses with PBS, cells were permeabilized with 0.5% TritonX-100 in PBS for 15 min at RT. Non-specific binding was blocked with 3% FBS and 0.1% TritonX-100 in PBS at RT for 30 min. Next, cells were incubated with primary antibodies in blocking buffer for 2 h at room temperature. The antibodies used included anti-Nestin antibody (R&D Systems, Minneapolis, MN, USA, Cat#MAB1259), Anti SOX2 Antibody (Abcam, Cambridge, UK, Cat#AB97959), Anti GFAP antibody (Millipore, Bedford, MA, USA, Cat#MB360), Anti DCX antibody (Millipore, Cat#AB5910, or Santa Cruz Biotechnology, Paso Robles, CA, USA, Cat#SC-8066), Anti-Tuj1 antibody (Promega, Cat#G7121) as well as Anti-BrdU antibody (Abcam, Cat#Ab1893). After rinsing the cells three times with PBS, they were incubated with secondary antibodies (Biotium, Inc., Fremont, CA, USA) containing DAPI (Dojindo Molecular Technologies, Kumamoto, Japan, Cat#D523) at RT for 1.5 h. Image acquisition was performed using an InCell Analyzer 6000 (Cytiva, Marlborough, MA, USA) or FV1200 (Olympus Corporation, Tokyo, Japan). Data analysis for nuclei counting and BrdU^+^ cell counting was performed using InCell Investigator Developer Toolbox (1.9.2) software (Cytiva).

### 4.4. Pharmacokinetic Analysis

The dosing solution was prepared in 0.5% methylcellulose in water. Lead-238 was administered at a dose of 20 mg/kg orally to 7-week-old normal male rats (Sprague-Dawley rats; three animals for each dose group). Blood samples were collected at the following scheduled time points: 15 and 30 min, and 1, 2, 4, 6, and 24 h. The plasma concentrations were determined by liquid chromatography–tandem mass spectrometry (LC-MS/MS). Predicted plasma concentrations at 30 mg/kg were estimated assuming dose-proportional pharmacokinetics. Prediction of brain concentrations was performed by multiplying the measured total plasma concentrations by the brain-to-plasma partition coefficient (Kp,total). The Kp,total value was obtained from a separate pharmacokinetic experiment in rats following oral administration of the test compound at 2 mg/kg, in which total drug concentrations in plasma and whole brain were quantified at 1 h post-dose. Predicted brain concentrations at 20–30 mg/kg were calculated as:

Predicted Brain Concentration (nM) = Plasma Concentration (nM) × K_p_.brain

### 4.5. In Vivo Pharmacological Evaluation

Male Wistar rats were obtained from Japan SLC Co., Ltd. (Shizuoka, Japan). They were housed in groups of 3–5 per cage in a controlled environment (temperature: 23 ± 2 °C; humidity: 55 ± 10%) with a 12 h light/dark cycle. Animals had ad libitum access to standard chow (CE2; Japan Clea, Tokyo, Japan) and filtered water. All animals were quarantined and acclimatized for one week prior to experimentation. All procedures were reviewed and approved by the Institutional Animal Care and Use Committee of Sumitomo Pharma Co., Ltd. To evaluate the effects of a test compound on neural stem cell proliferation, rats were orally administered Lead-238 (30 mg/kg) or vehicle twice daily for 6 consecutive days. In parallel, BrdU (50 mg/kg) was administered intraperitoneally twice daily to label proliferating cells.

### 4.6. Tissue Preparation and DAB-Based BrdU Staining in Rat Brain

Following the final administration, animals were euthanized and transcardially perfused with 4% paraformaldehyde (PFA). The brains were then collected and post-fixed by immersion fixation in 4% PFA at 4 °C for 48 h. Fixed tissues were transferred to phosphate-buffered saline (PBS) and processed for paraffin embedding. Brain regions, including the subventricular zone (SVZ; Bregma +1.0 mm to −0.5 mm) and hippocampus (Bregma −3.5 mm to −5.5 mm), were dissected according to a predefined cutting scheme. Paraffin embedding was performed using an automated embedding system, and 6-μm-thick sections were prepared using a rotary microtome. A total of 20 non-contiguous sections were collected per brain and used for immunohistochemical staining. Immunohistochemical staining for BrdU was performed using an automated Bond RX system (Leica Biosystems, Wetzlar, Germany). Antigen retrieval was conducted using Bond Epitope Retrieval Solution 1 (Leica Biosystems, Cat#AR9961). Endogenous peroxidase activity was blocked using Peroxidase Block (BioGenex Laboratories, Fremont, CA, USA, Cat#HK1115K-GP). Sections were incubated with a mouse monoclonal anti-BrdU antibody (clone Bu20a, Dako, Glostrup, Denmark; M0744; 2.62 μg/mL, 1:100 dilution) for 50 min at room temperature, followed by detection using a One-Step Polymer-HRP system (BioGenex Laboratories, Cat#HK595-50K). Visualization was achieved using DAB substrate (Super Sensitive™ DAB, BioGenex Laboratories, Cat#B-HK542XAK), and counterstaining was performed with hematoxylin. All sections were mounted with the cortical side oriented upward, and stained slides were submitted for histological evaluation. BrdU-positive nuclei in the SVZ and hippocampal dentate gyrus were quantified using Aperio ImageScope (v12.4.6.5003, Leica, Ellicott, MD, USA). Regions of interest were manually defined, and positive nuclei were identified based on intensity thresholding. Counts were confirmed by visual inspection.

### 4.7. Double Immunofluorescence Staining for BrdU/Nestin and BrdU/DCX in SVZ

Double immunofluorescence staining was performed to identify proliferating neural stem/progenitor cells and immature neurons in the SVZ. All staining and imaging procedures were conducted by Biopathology Institute Co., Ltd. (Kunisaki City, Oita Prefecture, Japan). Paraffin-embedded brain sections were first deparaffinized, rehydrated through graded ethanol, and rinsed in distilled water. After washing with Tris-buffered saline (TBS), antigen retrieval was performed using citrate buffer (pH 6.0) in an autoclave at 120 °C for 10 min. Sections were then washed again in TBS and incubated with a primary antibody against BrdU (Abcam, Cat#ab1893; 1:500 dilution) at 4 °C for 1 h. After TBS washing, Alexa Fluor 594-conjugated donkey anti-sheep IgG (Invitrogen, Carlsbad, CA, USA, Cat#A11016; 1:100 dilution) was applied at room temperature for 1 h. Subsequently, sections were incubated with a second primary antibody depending on the target marker. For Nestin staining, a mouse monoclonal anti-Nestin antibody (Abcam, Cat#ab6142; 1:50 dilution) was used to label neural stem/progenitor cells. For Doublecortin (DCX) staining, a rabbit polyclonal anti-DCX antibody (Abcam, Cat#ab18723; 1:1000 dilution) was used to label immature neurons. After TBS washing, the appropriate secondary antibody was applied: Alexa Fluor 488-conjugated goat anti-mouse IgG (Invitrogen, Cat#A11029; 1:100 dilution) for Nestin, and Alexa Fluor 488-conjugated goat anti-rabbit IgG (Invitrogen, Cat#A11034; 1:100 dilution) for DCX. Finally, sections were washed and mounted using SlowFade Gold Antifade reagent with DAPI (Invitrogen, Cat#S36938). Fluorescence images were acquired using a high-resolution microscope, and representative fields from SVZ regions were selected for quantitative analysis. ROIs were manually defined based on anatomical landmarks. Quantification of BrdU/Nestin and BrdU/DCX double-positive cells was performed using ImageJ software1.54g (NIH, Bethesda, MD, USA), applying consistent thresholding and particle analysis parameters. Sections or regions with poor staining quality, tissue damage, or processing artifacts were excluded from the analysis.

### 4.8. Statistical Analyses

Statistical analyses and graph preparation were performed using Prism 6.0 (GraphPad Software Inc., San Diego, CA, USA). Data are presented as the mean, and each bar indicates the standard deviation. Data normality was assessed using the Shapiro–Wilk test or visually using histograms for small sample sizes. Depending on the result, unpaired *t*-tests (for parametric data) were used for comparisons between two groups. Dunnett’s multiple comparison method was used for comparing multiple treatments to a control group. *p* values, where shown, indicate significance between each group (*, *p* < 0.05; **, *p* < 0.01; ***, *p* < 0.001; ****, *p* < 0.0001). Dose–response curves were fitted using a four-parameter logistic (4PL) model (GraphPad Prism 6.0). For ATP-based proliferation, the maximal effect (Emax) was constrained to the measured plateau response, which represented the highest reproducible activity. EC_50_ values were extracted from the fitted models.

## 5. Conclusions

In this study, we established a human neural stem cell–based phenotypic screening platform under growth factor–free conditions and identified Lead-238 as a small molecule with pronounced intrinsic neurogenic activity. Lead-238 selectively enhanced neurogenic activity in the subventricular zone in vivo, as indicated by increased BrdU/Nestin- and BrdU/DCX-positive cells, without detectable effects in the subgranular zone, while achieving sufficient brain exposure. Its activity could not be readily attributed to established neurogenic pathways, supporting a distinct pharmacological profile. These findings highlight the utility of growth factor–free neural stem cell screening for identifying compounds that enhance adult neurogenic activity and warrant further investigation of the molecular mechanism and translational potential of Lead-238.

## Figures and Tables

**Table 1:** Predicted Brain Concentrations of Lead-238 in Rats Following Oral Administration.

Time (h)	Plasma Conc. (20 mg/kg, nM)	Estimated Brain Conc. * (20 mg/kg, nM)	Estimated Brain Conc. ** (30 mg/kg, nM)
0.25	6.34	116	174.0
0.5	36.7	671	1006.5
1	93.7	1714	2571.0
2	81.7	1495	2242.5
4	34.4	629	943.5
6	15.9	291	436.5
24	0.403	7.37	11.1

* Brain concentrations were estimated using a K_p_ value of 18.3, calculated from 2 mg/kg 1-h experimental data. ** Brain concentrations at 30 mg/kg were extrapolated from the 20 mg/kg estimated values, assuming linear pharmacokinetics.

## Data Availability

The original contributions presented in this study are included in the article/Supplementary Material. Further inquiries can be directed to the corresponding author.

## Supplementary Materials

The following supporting information can be downloaded at: https://www.mdpi.com/article/10.3390/ph19040536/s1, Figure S1: Characterization of human neural stem cells for neurogenic compound discovery; Figure S2: Assay quality assessment for High-throughput screening using hNSCs; Figure S3: Counter assay of hit compounds in HT-29; Figure S4: Predicted brain concentration–time profiles of Lead-238; Figure S5: Molecular docking analysis of PKC C1 domains; Figure S6: Low-magnification anatomical overview of the lateral ventricles (LV) and the adjacent subventricular zone (SVZ); Table S1: Physicochemical property ranges of Lead-238; Table S2: Binding activity of Lead-238 on PKCα; Table S3: Agonist activity of Lead-238 on Dopamine and Serotonin receptors.

## Abbreviations

The following abbreviations are used in this manuscript:

ADME	Absorption, Distribution, Metabolism, and Excretion
ATP	Adenosine Triphosphate
BrdU	Bromodeoxyuridine
DAB	3,3′-diaminobenzidine
DAPI	4′,6-Diamidino-2-Phenylindole
DCX	Doublecortin
DMSO	Dimethyl Sulfoxide
EGF	Epidermal Growth Factor
EGFR	Epidermal Growth Factor Receptor
FGF-2	Fibroblast Growth Factor 2
GPCR	G Protein-Coupled Receptor
HRP	Horseradish Peroxidase
LC–MS/MS	Liquid Chromatography–Tandem Mass Spectrometry
NSC	Neural Stem Cell
PBS	Phosphate-Buffered Saline
SVZ	Subventricular Zone
SGZ	Subgranular Zone
CNS	Central Nervous System
PKC	Protein Kinase C
RT	Room Temperature
ROI	Region of Interest
