# Supplementary material for: Discovery of a Novel Compound Enhancing SVZ Neurogenic Effects via Human Neural Stem Cell-Based Phenotypic Screening
Source: Pharmaceuticals (Basel). 2026 Mar 26;19(4):536. doi: 10.3390/ph19040536 (PMC13118763; doi:10.3390/ph19040536)
Supplement: Supplementary file 1 [file pharmaceuticals-19-00536-s001.zip › pharmaceuticals-4200734-supplementary.pdf]

Supplementary Materials

Discovery of a Novel Compound Enhancing SVZ Neurogenic Effects via Human Neural Stem Cell-Based Phenotypic Screening

Atsushi Nakane †,‡, Katsushi Kitahara †, Riku Fukushima, Tetsuro Nariai §, Kazuto Yamazaki ‖ and Hidetaka Nagata *

Sumitomo Pharma Co., Ltd. 1–98, Kasugade-naka 3-chome, Konohana-ku, Osaka 554-0022, Japan; atsushi.nakane@racthera.co.jp (A.N.); katsushi.kitahara@sumitomo-pharma.co.jp (K.K.); riku.fukushima@sumitomo-pharma.co.jp (R.F.);

* Correspondence: hidetaka.nagata@sumitomo-pharma.co.jp

† These authors contributed equally to this work.

‡ Current address: Kobe Research Center, RACTHERA Co., Ltd., Kobe 650-0047, Japan.

§ Current address: Sumitomo Chemical Co., Ltd., Osaka 554-0022, Japan.

‖ Current address: Independent Researcher, Osaka 530-0001, Japan.

Supplementary Methods

Counter Assay using HT-29

HT-29 were cultured in McCoy's 5A supplemented with 10% fetal calf serum (FCS) and penicillin-streptomycin. To evaluate cell proliferation under cancer stem cell-like conditions, cells were seeded in Ultra Low Attachment 96-well plates (Corning, Corning, NY, USA, Cat#3474) at a density of 350 cells/well for HT-29 to promote spheroid formation. On Day 4, cell proliferation was assessed using the CellTiter-Glo Luminescent Cell Viability Assay (Promega, Madison, WI, USA Cat#G7570) according to the manufacturer's instructions. Luminescence was measured using an Envision plate reader (Revvity, Waltham, MA, USA).

In vitro PKC binding assay

The binding assay was performed using a His-tag capture approach adapted from previously reported protocol [46]. Briefly, the PKCα C1B domain (residues 96–156) was prepared in buffer A (25 mM HEPES, pH 7.5; 100 mM NaCl; 0.5 mM TCEP; 0.5% DMSO; 20 μM $ZnSO_4$) at 0.05 mg/mL. Lead-238 was diluted from a 10 mM DMSO stock to a final concentration of 80 μM (2% DMSO). His-tag magnetic beads were diluted 10-fold and incubated with protein and compound solutions (10 μL each) in Corning 3788 plates (total 30 μL) for 2 h at room temperature with gentle shaking. After washing with buffer A, bound compounds were eluted with 80% methanol/1% acetic acid, processed using Oasis PRiME HLB plates, and analyzed by LC–MS (LTQ Orbitrap, C18 column; mobile phase: 20% water/80% methanol + 0.5% formic acid). Peaks corresponding to $[M+H]^+$ were extracted within ±10 ppm. Binding was considered positive when the signal exceeded 2 × SD above the mean of control samples lacking PKCα (n = 5).

Molecular docking analysis

The molecular modeling analysis was carried out using Molecular Operating Environment (MOE) software (Chemical Computing Group Inc., Montreal, QC, Canada). Human PKCs C1 domain amino acid sequences were obtained from UniProt database. The three-dimensional structures and conformations of the proteins were predicted using the AlphaFold2 [44] through ColabFold [45]. The protein structures were prepared by the addition of hydrogens using the option "Protonate 3D" and energy minimization using AmberEHT forcefield. The compounds were constructed in MOE using the builder module, prepared, and then collected in a database. Hydrogens were also added to the atom of the constructed compounds and the partial charges were calculated. The prepared compounds were docked to each of the proteins, and the best binding pose was selected based on the GBVI/WSA dG score. The docking protocol was validated using the co-crystal structure of the rat PKCδ C1B domain (PDB ID: 7LCB) by reproducing the binding pose of the co-crystallized ligand, Prostratin, within its binding pocket. The obtained root mean square deviation (RMSD) was less than 0.2 Å.

GPCR functional assay

GPCR functional assay was performed according to previous report [47]. In brief, calcium mobilization of Lead-238 (1 μM) was measured by FDSS7000EX System (Functional Drug Screening System: Hamamatsu Photonics K.K., Shizuoka, Japan). Agonistic activity of each receptor transiently expressed in CHO cells is indicated as percentage of maximum response of each ligand. In the case of Gi- and Gs-coupled receptors, Gα16 proteins were also co-expressed to increase the sensitivity of calcium signaling.

Supplementary Figures

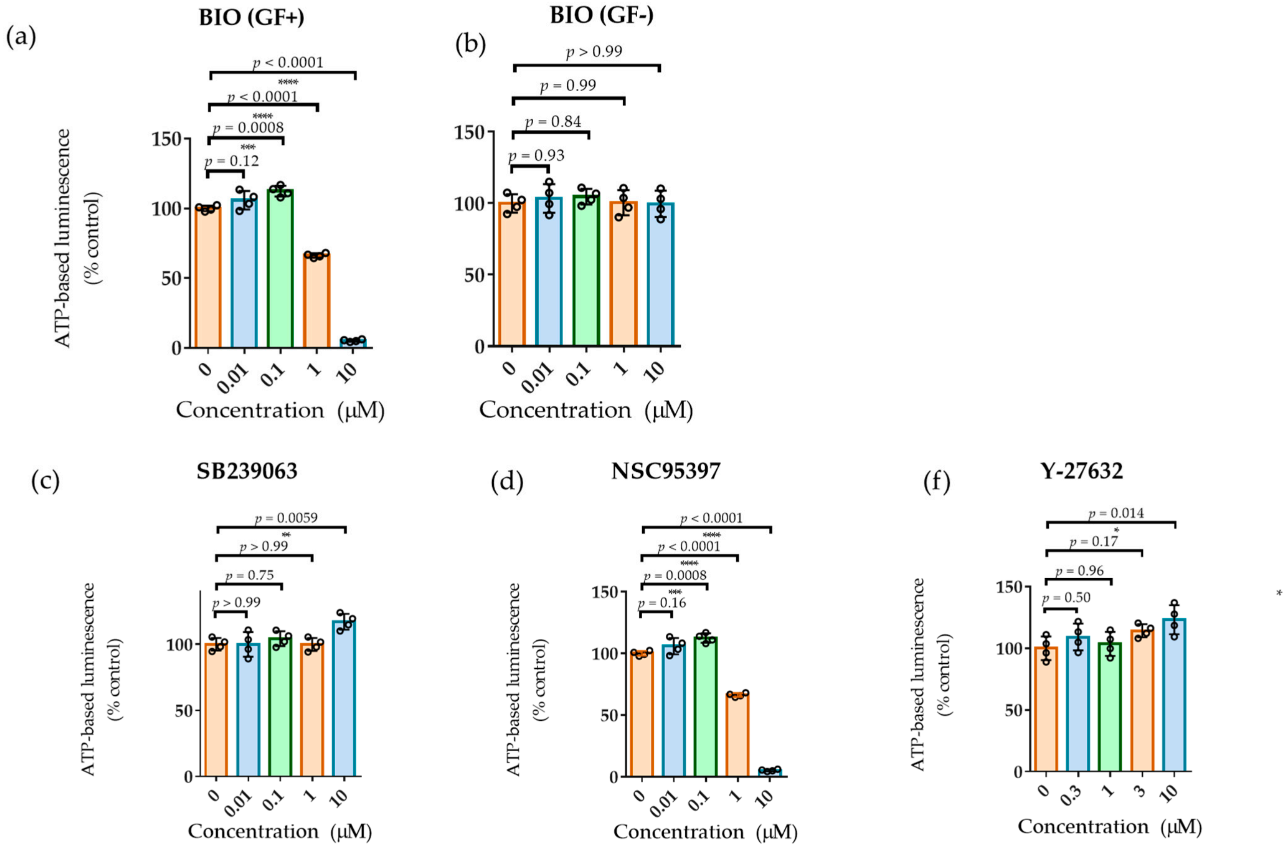

Figure S1. Characterization of human neural stem cells (hNSCs) for neurogenic compound discovery.

(a,b) Dose-dependent effects of BIO, a Wnt pathway activator, on hNSC proliferation under growth factor-supplemented (GF+) and growth factor-free (GF⁻) conditions. (c,d,e) Dose-dependent effects of SB239063 (p38 MAPK inhibitor) , NSC95397 (Cdc25 phosphatase inhibitor) and Y-27632 (ROCK inhibitor) on hNSC proliferation under growth factor-free conditions. Data are presented as mean ± SD, $n = 4$. Statistical significance: *$p < 0.05$, **$p < 0.01$, ***$p < 0.001$, ****$p < 0.0001$ (Dunnett's multiple comparisons test).

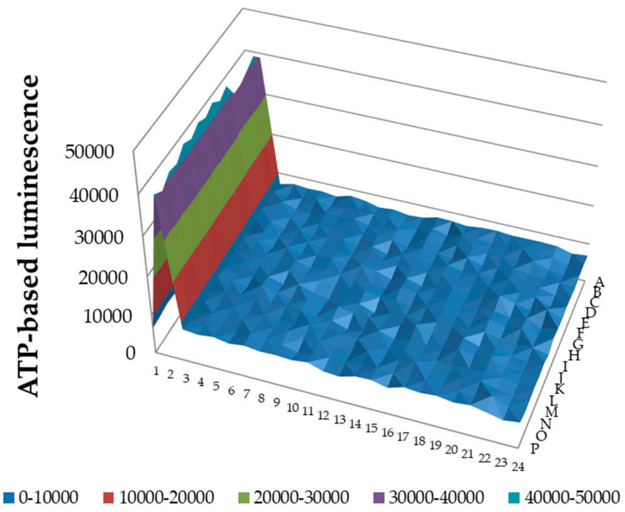

Figure S2. Assay quality assessment for High-throughput screening using hNSCs

Luminescence measurements across a 384 well plate. Only the second column received growth factor (GF) supplementation, while the first column contained DMSO only (negative control). The Z′ factor was calculated based on luminescence values from these controls and yielded a value of 0.826. The Z′ factor was calculated using the standard formula:

Z' = 1 − 3 × (σp + σn) / ◎μp − μn◎,

where μp and σp represent the mean and SD of the GF-supplemented wells (positive control), and μn and σn represent those of the DMSO wells (negative control).

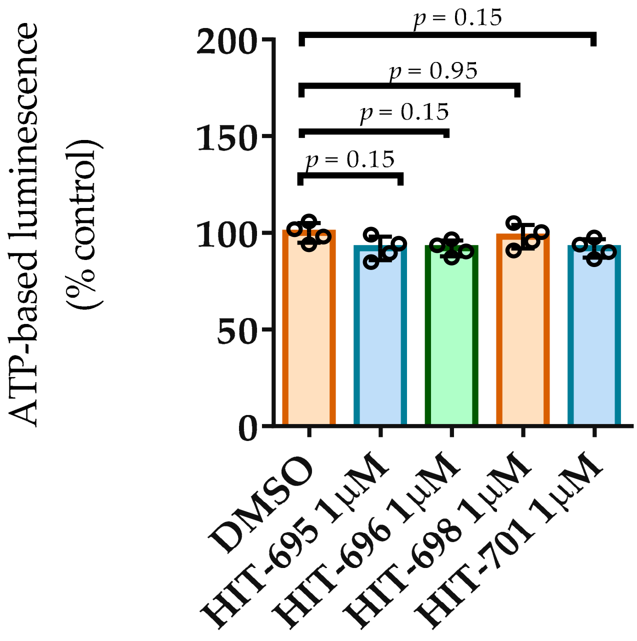

Figure S3 Counter assay of hit compounds in HT-29

Hit compounds identified from the hNSC phenotypic screening were tested for proliferative effects in a non-neural cell line (HT-29) to assess neural selectivity. Each compound was evaluated at 1μM, the concentration that showed the highest proliferative activity in hNSCs. Individual data points are shown together with mean ± SD (n = 4). Exact p-values obtained from Dunnett's multiple-comparison test are directly annotated on each graph. No significant increase in ATP levels was observed.

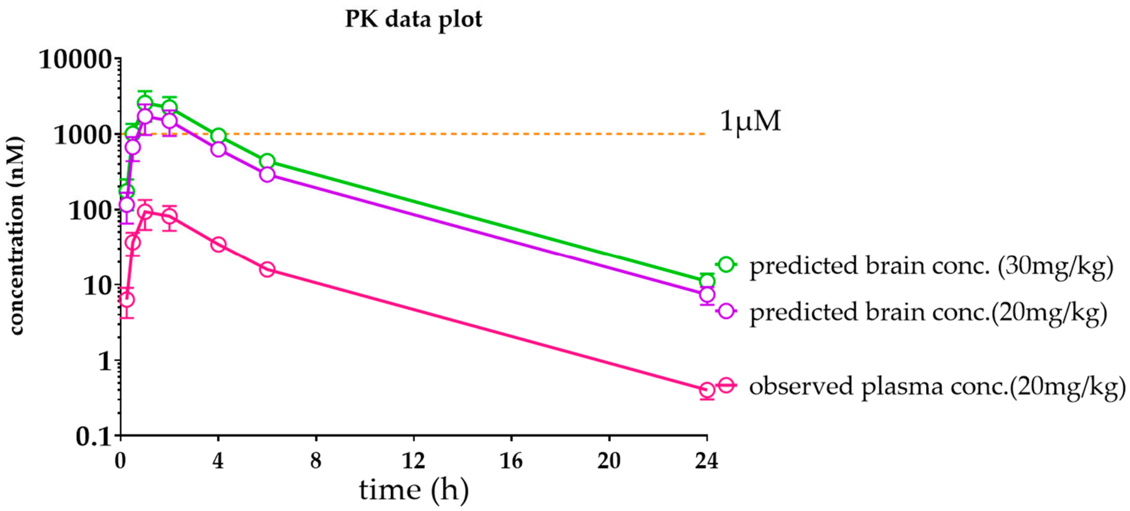

Figure S4. Predicted brain concentration–time profiles of Lead-238.
Observed plasma concentrations (20 mg/kg, oral) and predicted brain concentrations at 20 and 30 mg/kg are plotted with time (h) on the x-axis and concentration (nM) on the y-axis (*n* = 3 per time point). Data are presented as mean ± SD. Predicted brain concentrations were calculated as plasma concentration × Kp,brain and the detailed prediction procedure is described in the Methods section of the main manuscript. Standard deviations of predicted brain concentrations were obtained by error propagation using the equation:

the equation: $SD_B = B\sqrt{\left(SD_P/P\right)^2 + \left(SD_{K_p}/K_p\right)^2}$

where B is the predicted brain concentration, P is the plasma concentration, SD_P is the SD of the plasma measurement, and SD_{Kp} is the SD of the brain-to-plasma partition coefficient. The yellow dotted line represents a brain concentration of 1 μM for reference.

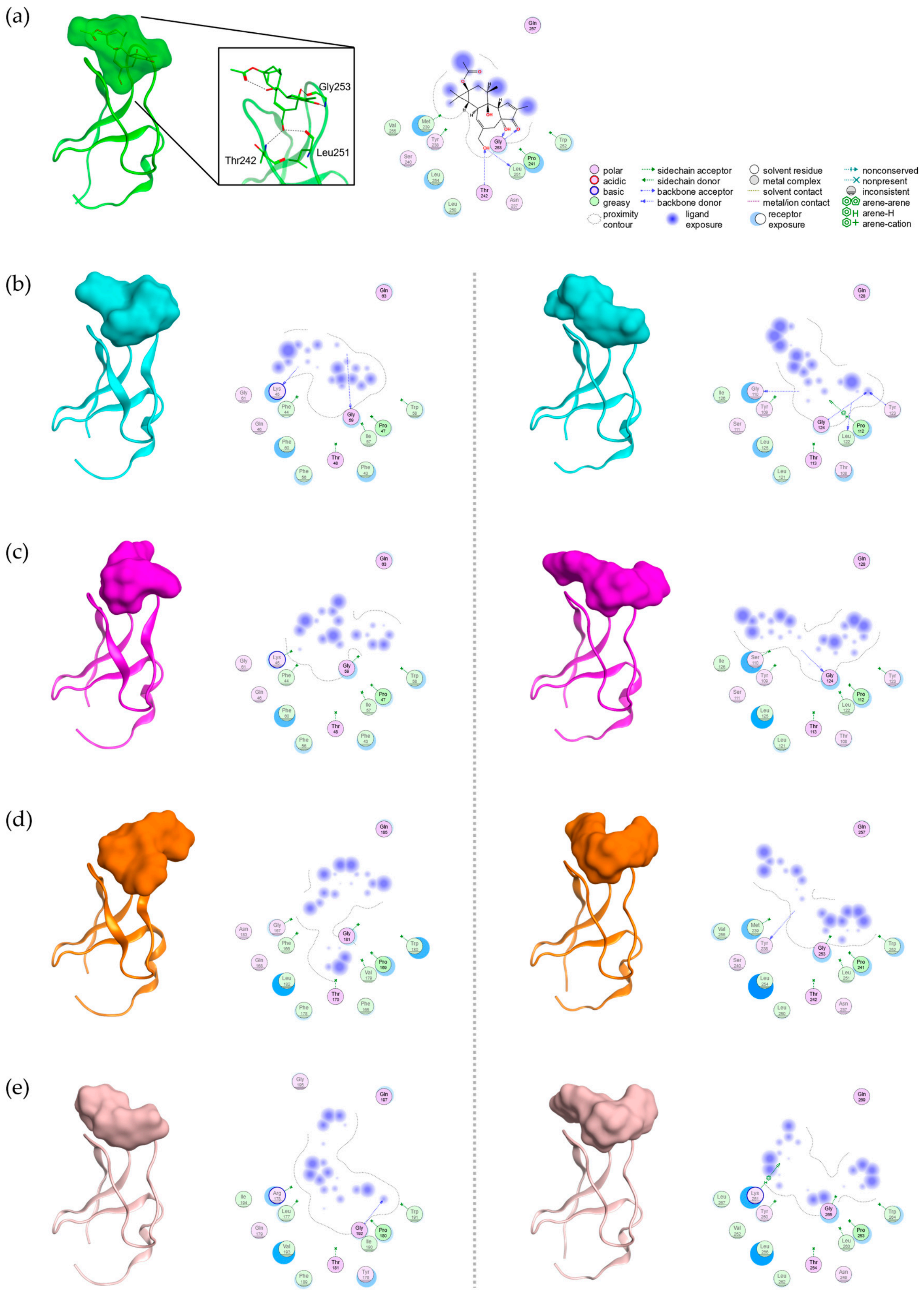

Figure S5. Molecular docking analysis of PKC C1 domains

(a) Crystal structure of the rat PKCδ C1B domain complexed with Prostratin (PDB ID: 7LCB). The

right schematic diagram shows interactions between the amino acid residues and the ligand. The ligand forms several hydrogen bonds with the backbone atoms. The hydrogen bonds between Thr242 and ligand plays a critical role in ligand binding. (b-e) Docking models and interaction diagrams of Lead-238 for human PKC C1 domains (PKCα, PKCβ, PKCδ and PKCε). Each Left panel shows the C1A domain, and right panel shows the C1B domain. In all docking models, the compound does not form hydrogen bonds with the conserved threonine residue at the bottom of the binding pocket and instead occupies a shallow region within the pocket, suggesting that its binding to these C1 domain is negligible.

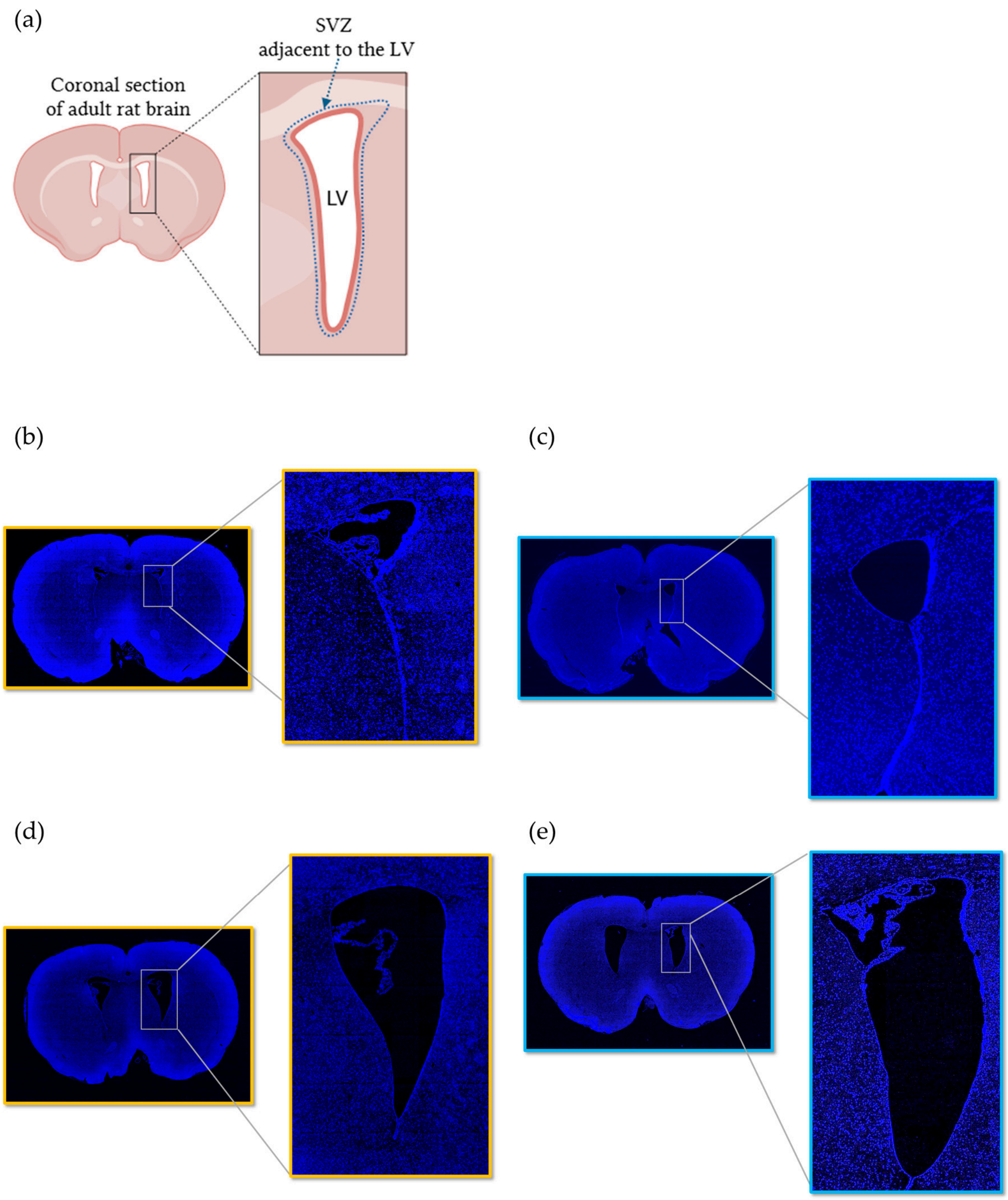

Figure S6. Low-magnification anatomical overview of the lateral ventricles (LV) and the adjacent subventricular zone (SVZ)

(a) A schematic coronal section of the adult rat brain illustrates the anatomical location of the SVZ along the LV walls. (b-e) Low-magnification DAPI images (blue) of representative coronal brain sections from each experimental group are shown, depicting the full extent of the left and

right lateral ventricles. For each section, the boxed region indicates the SVZ area corresponding to the high-magnification images shown in Figure 7. Specifically, Fig. S6b, S6c, S6d, and S6e correspond to Fig. 7b, 7c, 7e, and 7f, respectively. These overview images provide anatomical context for the SVZ-focused high-magnification panels in Figure 7. Images were acquired using a virtual slide-scanning system (equivalent to 20× magnification).

Supplementary Tables

Table S1 Physicochemical property ranges of Lead-238

This table summarizes key predicted physicochemical properties of Lead-238, including molecular weight, lipophilicity, hydrogen-bond donor/acceptor counts, aromatic ring count, and topological polar surface area (TPSA). These ranges are provided as alternative compound information

**Physicochemical property**	**Ranges**
Molecular Weight	350-400
Lipophilicity (cLogP)	3.0-3.5
Number of Hydrogen Bond Donor	1-3
Number of Hydrogen Bond Acceptor	3-6
Number of Aromatic Rings	1-3
Topological Polar Surface Area (TPSA)	50-70

Table S2 Binding activity of Lead-238 on PKCα

Binding affinity was assessed using a His-tag capture assay followed by LC–MS analysis. Values represent fold-change in peak area relative to control samples lacking PKCα. Positive controls (phorbol 12,13-dibutylate and prostratin) showed significant enrichment, whereas Lead-238 exhibited no detectable binding under the tested conditions. Statistical significance was indicated as follows: *p < threshold for >2 SD above control; **p < threshold for >3 SD above control (n = 5).

Protein	Phorbol 12,13-dibutylate	Prostratin	Lead-238
KPCA (96-156)	4.0**	2.2*	-1.6

Table S3 Agonist activity of Lead-238 on Dopamine and Serotonin receptors

GPCR functional assays were performed using calcium mobilization in CHO cells transiently expressing the indicated receptors. Columns from left to right: GPCR, Ligand (concentration), number of replicates, and % Agonist Activity. Responses are expressed as % of the maximal response induced by the reference ligand for each receptor. Lead-238 (1 μM) showed no meaningful agonist activity across dopamine D1–D5 and serotonin 5-HT_1–5-HT_7 receptor subtypes.

GPCR	Ligand	Rep.	% Agonist Activity
Dopamine D1	Dopamine (1 μM)	4	-2.76
Dopamine D2L	Dopamine (1 μM)	4	2.81
Dopamine D2S	Dopamine (1 μM)	4	0.63
Dopamine D3	Dopamine (1 μM)	4	3.48
Dopamine D4	Dopamine (1 μM)	4	15.07
Dopamine D5	Dopamine (1 μM)	4	-0.37
Serotonin 5-HT_{1A}	Serotonin (100 nM)	4	0.14
Serotonin 5-HT_{1B}	Serotonin (300 nM)	4	0.87
Serotonin 5-HT_{1E}	Serotonin (30 nM)	4	0.12
Serotonin 5-HT_{1F}	Serotonin (1 μM)	4	0.17
Serotonin 5-HT_{2A}	Serotonin (100 nM)	4	0.01
Serotonin 5-HT_{2B}	Serotonin (100 nM)	4	-0.08
Serotonin 5-HT_{2C}	Serotonin (100 nM)	4	0.2
Serotonin 5-HT_{4B}	Serotonin (100 nM)	4	0.13
Serotonin 5-HT_{5A}	Serotonin (100 nM)	4	2.01
Serotonin 5-HT_6	Serotonin (10 nM)	4	1.34
Serotonin 5-HT_{7B}	Serotonin (1 μM)	4	0.72

Supplementary References

44. Jumper, J.; Evans, R.; Pritzel, A.; Green, T.; Figurnov, M.; Ronneberger, O.; Tunyasuvunakool, K.; Bates, R.; Žídek, A.; Potapenko, A.; et al. Highly Accurate Protein Structure Prediction with AlphaFold. *Nature* **2021**, *596*, 583–589, doi:10.1038/s41586-021-03819-2.
45. Mirdita, M.; Schütze, K.; Moriwaki, Y.; Heo, L.; Ovchinnikov, S.; Steinegger, M. ColabFold: Making Protein Folding Accessible to All. *Nat Methods* **2022**, *19*, 679–682, doi:10.1038/s41592-022-01488-1.
46. Do Amaral, B.S.; De Moraes, M.C.; Cardoso, C.L.; Cass, Q.B. Affinity Selection Mass Spectrometry (AS-MS) for Prospecting Ligands in Natural Product Libraries. *Front. Nat. Prod.* **2025**, *4*, 1562501, doi:10.3389/fntpr.2025.1562501.
47. Sumiyoshi, T.; Enomoto, T.; Takai, K.; Takahashi, Y.; Konishi, Y.; Uruno, Y.; Tojo, K.; Suwa, A.; Matsuda, H.; Nakako, T.; et al. Discovery of Novel *N* -Substituted Oxindoles as Selective M_1 and M_4 Muscarinic Acetylcholine Receptors Partial Agonists. *ACS Med. Chem. Lett.* **2013**, *4*, 244–248, doi:10.1021/ml300372f.
